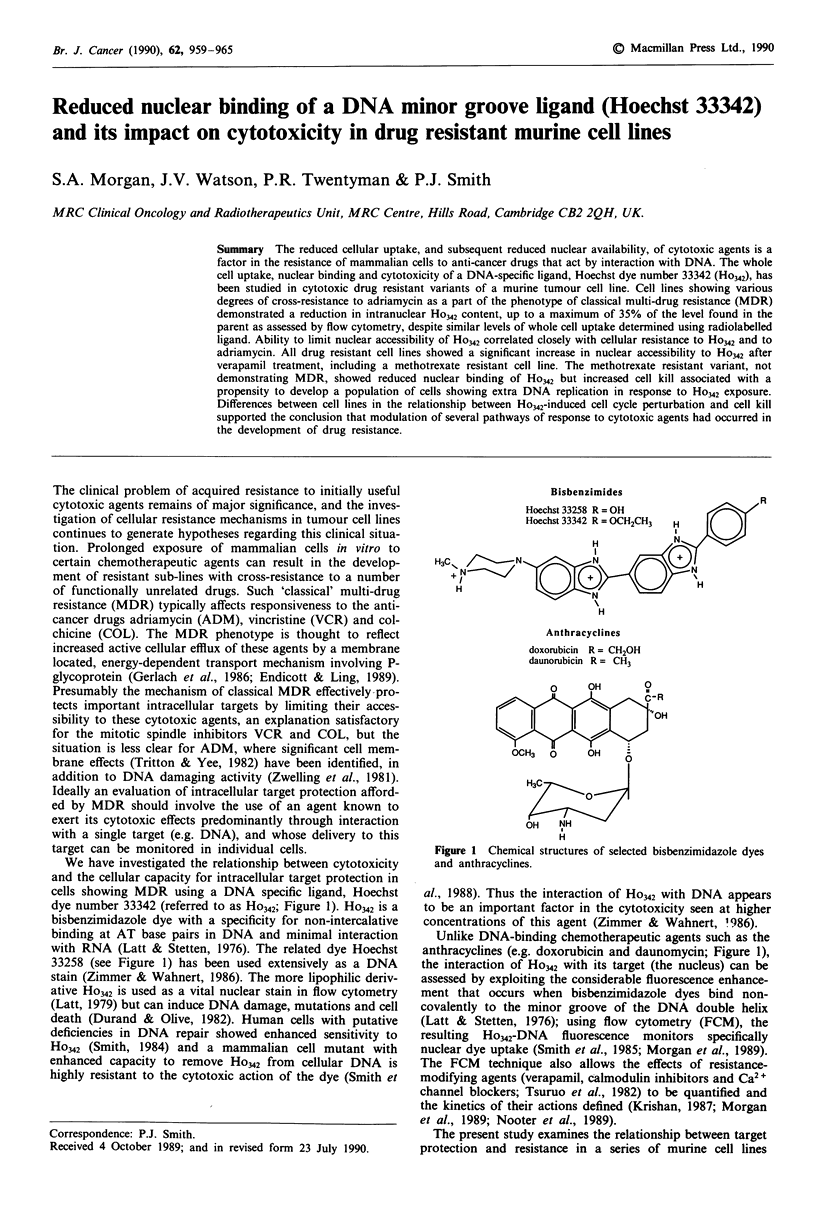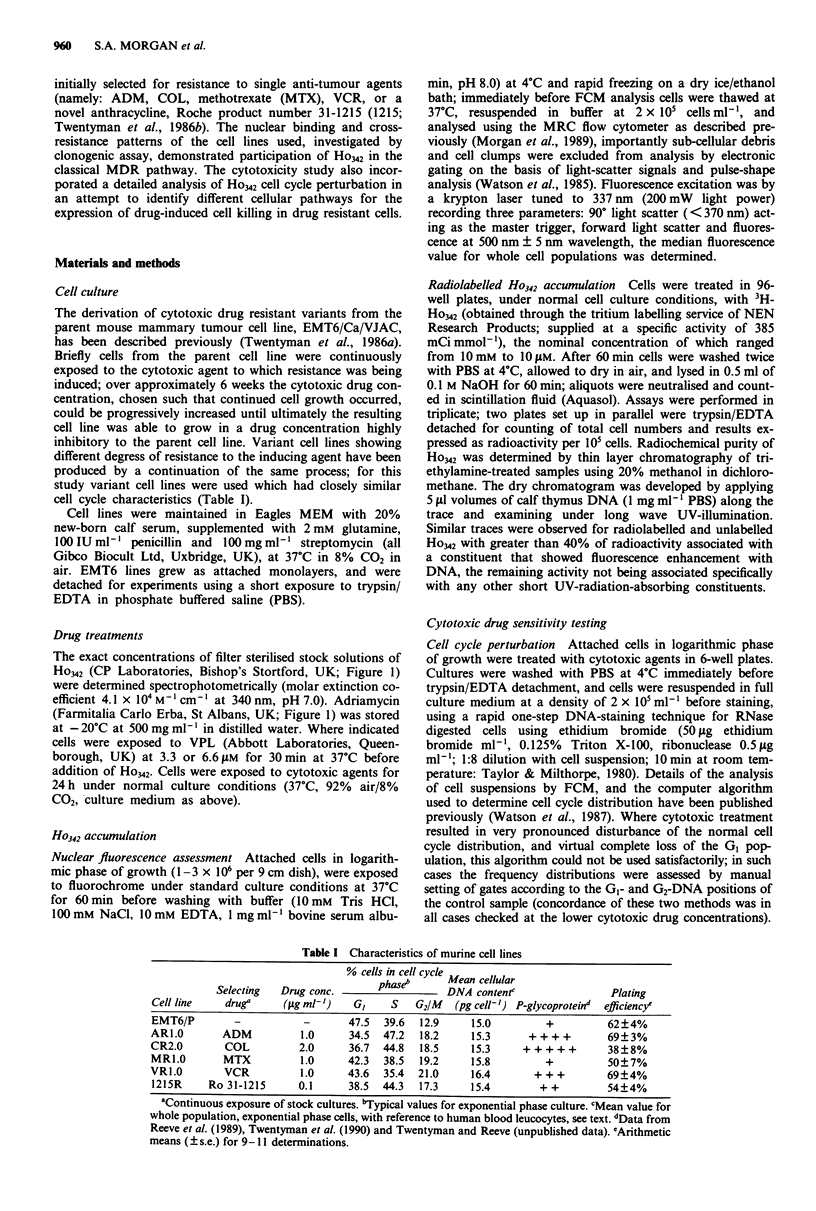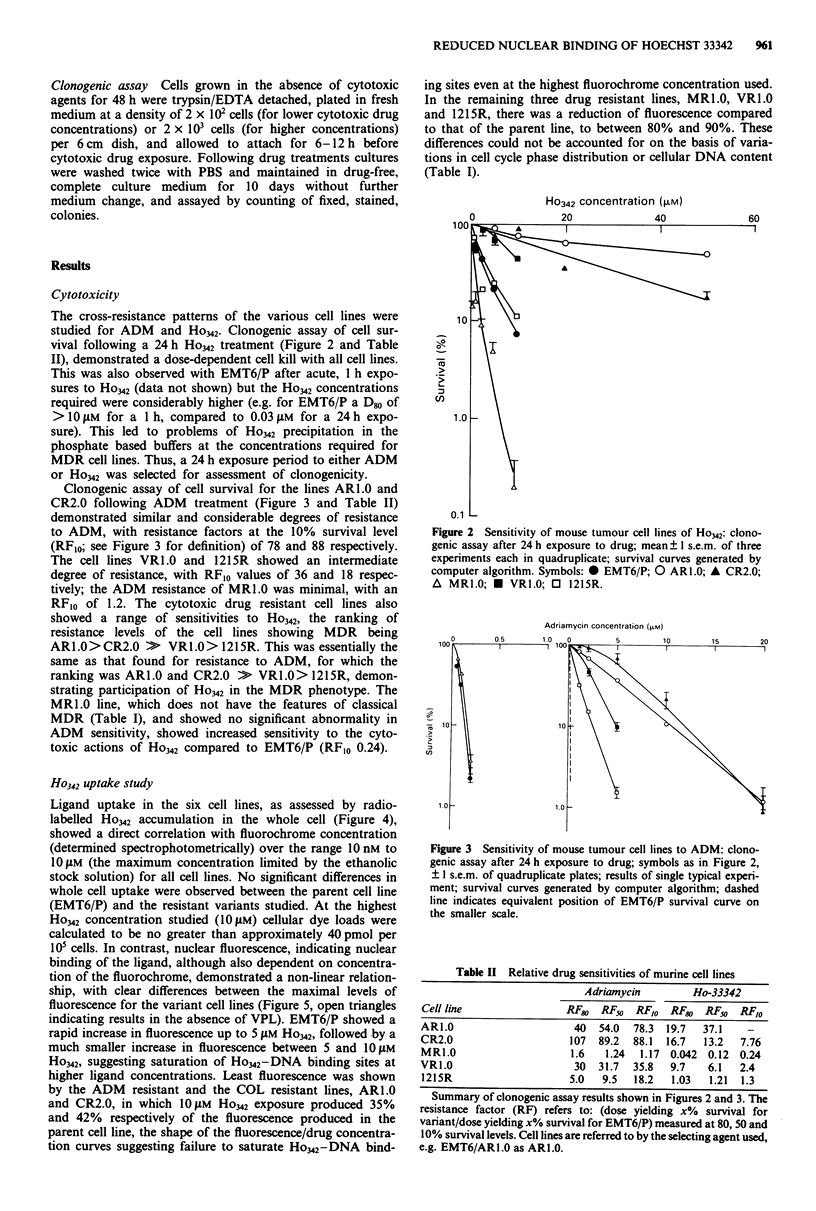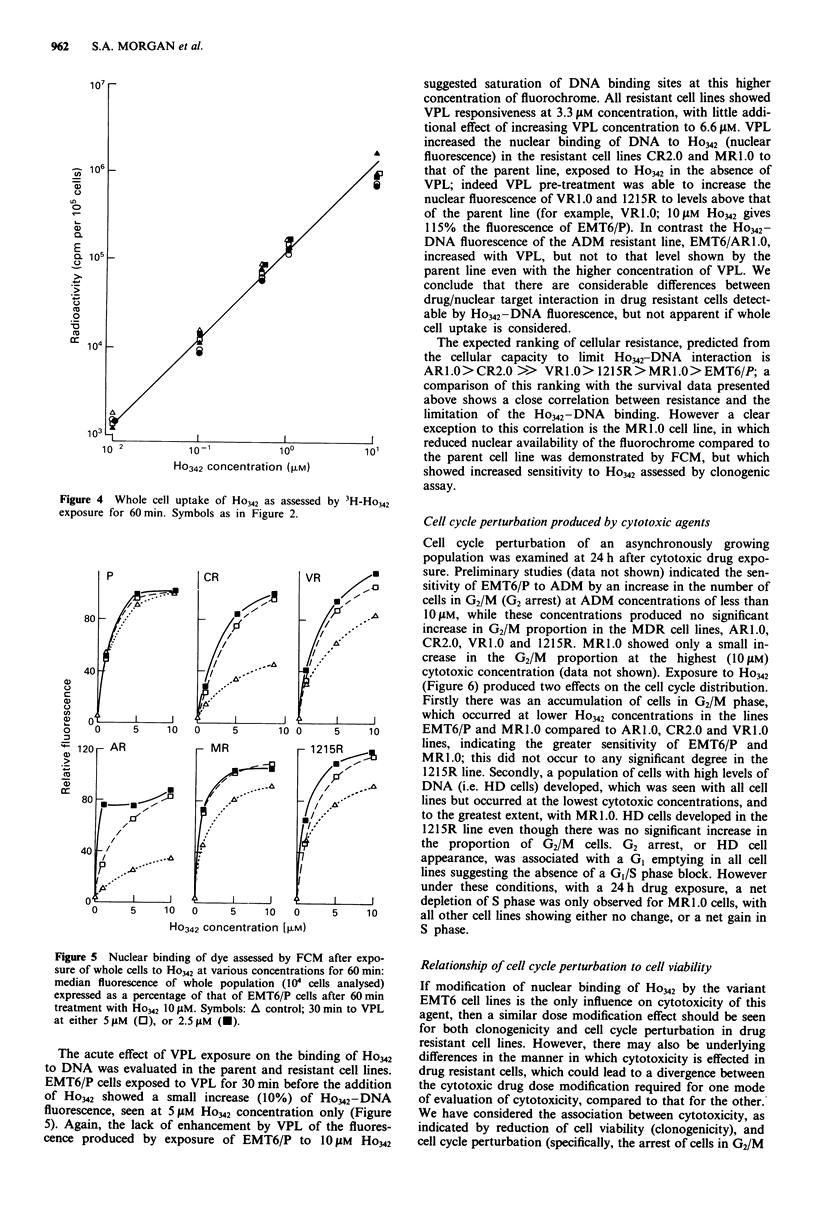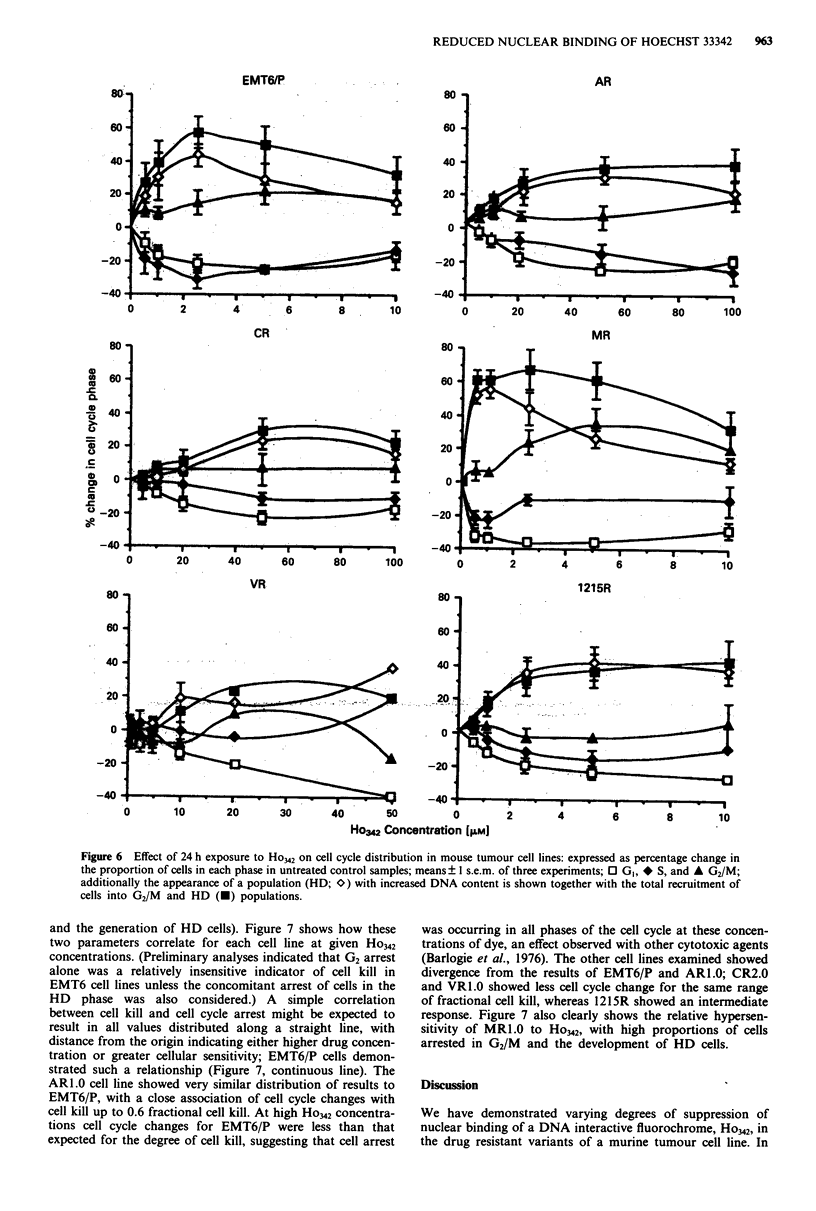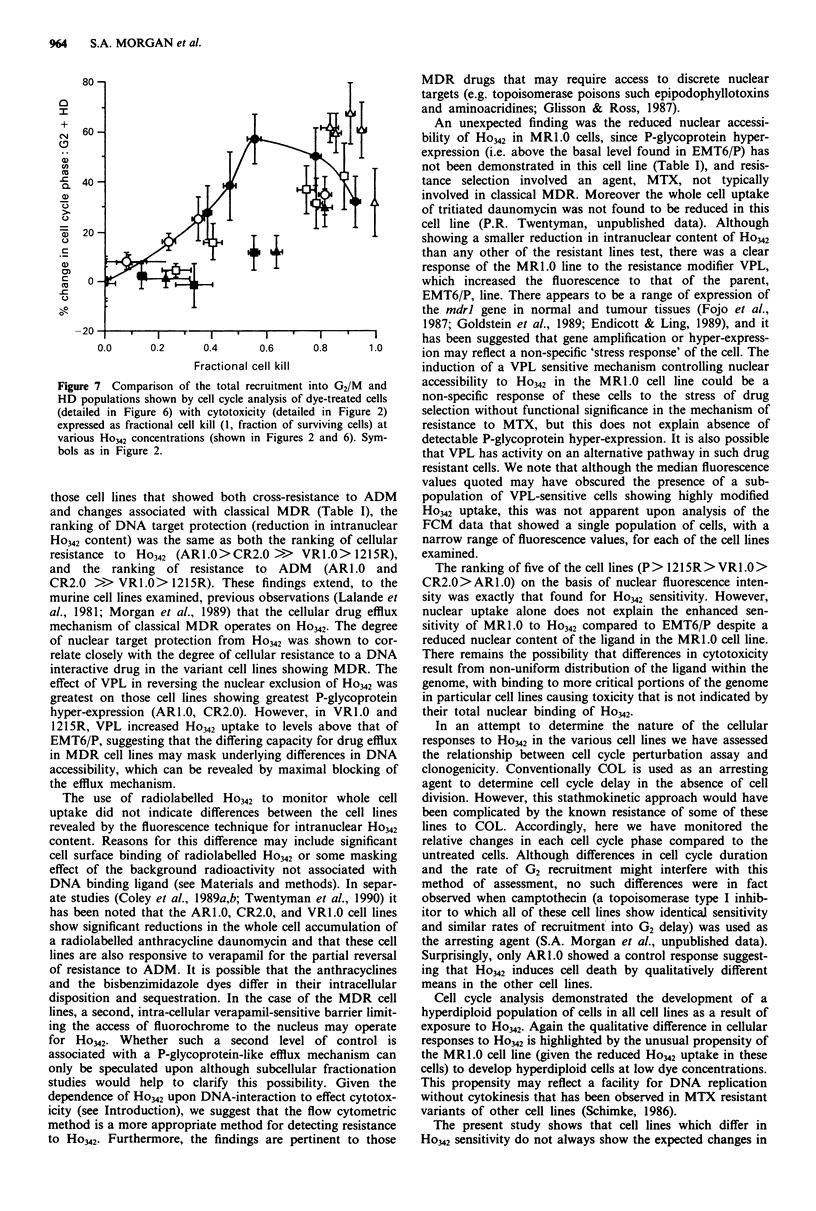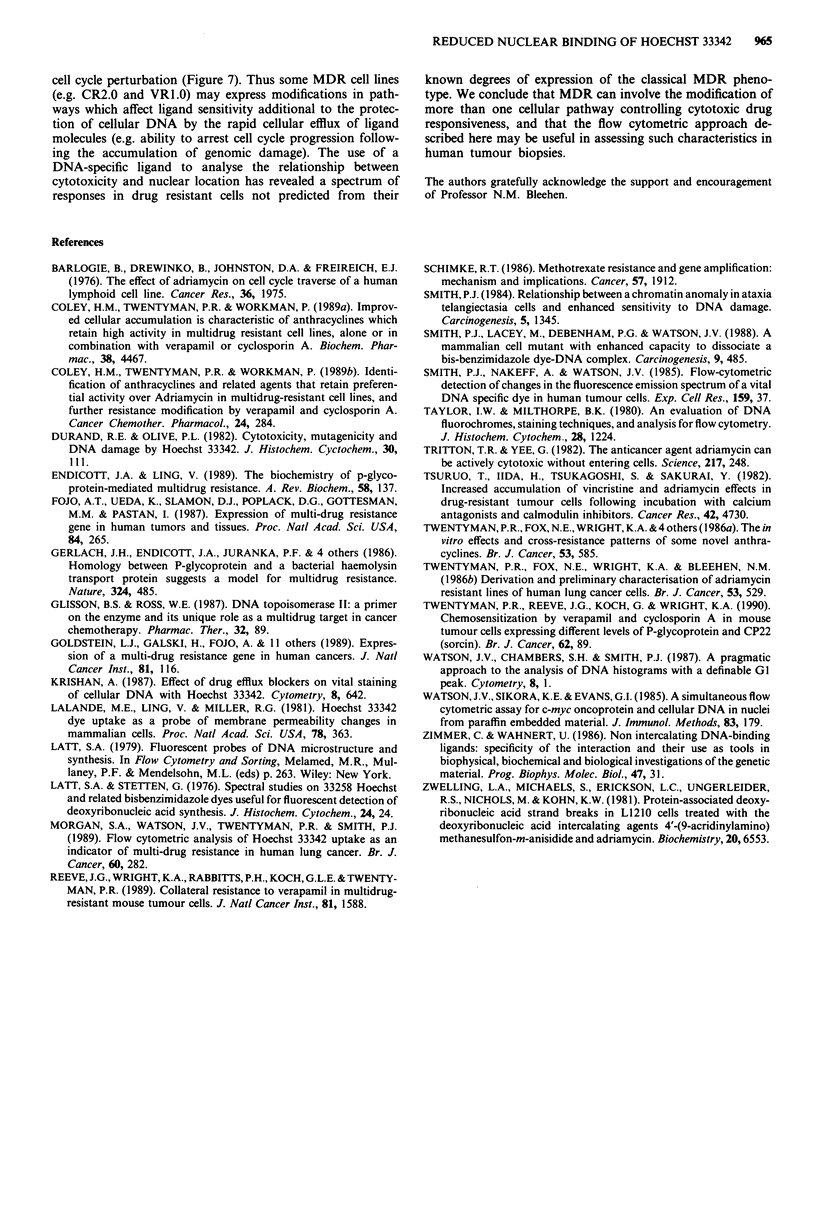# Reduced nuclear binding of a DNA minor groove ligand (Hoechst 33342) and its impact on cytotoxicity in drug resistant murine cell lines.

**DOI:** 10.1038/bjc.1990.417

**Published:** 1990-12

**Authors:** S. A. Morgan, J. V. Watson, P. R. Twentyman, P. J. Smith

**Affiliations:** MRC Clinical Oncology and Radiotherapeutics Unit, MRC Centre, Cambridge, UK.

## Abstract

The reduced cellular uptake, and subsequent reduced nuclear availability, of cytotoxic agents is a factor in the resistance of mammalian cells to anti-cancer drugs that act by interaction with DNA. The whole cell uptake, nuclear binding and cytotoxicity of a DNA-specific ligand, Hoechst dye number 33342 (Ho342), has been studied in cytotoxic drug resistant variants of a murine tumour cell line. Cell lines showing various degrees of cross-resistance to adriamycin as a part of the phenotype of classical multi-drug resistance (MDR) demonstrated a reduction in intranuclear Ho342 content, up to a maximum of 35% of the level found in the parent as assessed by flow cytometry, despite similar levels of whole cell uptake determined using radiolabelled ligand. Ability to limit nuclear accessibility of Ho342 correlated closely with cellular resistance to Ho342 and to adriamycin. All drug resistant cell lines showed a significant increase in nuclear accessibility to Ho342 after verapamil treatment, including a methotrexate resistant cell line. The methotrexate resistant variant, not demonstrating MDR, showed reduced nuclear binding of Ho342 but increased cell kill associated with a propensity to develop a population of cells showing extra DNA replication in response to Ho342 exposure. Differences between cell lines in the relationship between Ho342-induced cell cycle perturbation and cell kill supported the conclusion that modulation of several pathways of response to cytotoxic agents had occurred in the development of drug resistance.


*Br. J. Cancer* (1990), **62**, 959–965 © Macmillan Press Ltd., 1990

### Reduced nuclear binding of a DNA minor groove ligand (Hoechst 33342) and its impact on cytotoxicity in drug resistant murine cell lines

S.A. Morgan, J.V. Watson, P.R. Twentyman & P.J. Smith

*MRC Clinical Oncology and Radiotherapeutics Unit, MRC Centre, Hills Road, Cambridge CB2 2QH, UK.*

**Summary** The reduced cellular uptake, and subsequent reduced nuclear availability, of cytotoxic agents is a factor in the resistance of mammalian cells to anti-cancer drugs that act by interaction with DNA. The whole cell uptake, nuclear binding and cytotoxicity of a DNA-specific ligand, Hoechst dye number 33342 ($Ho_{342}$), has been studied in cytotoxic drug resistant variants of a murine tumour cell line. Cell lines showing various degrees of cross-resistance to adriamycin as a part of the phenotype of classical multi-drug resistance (MDR) demonstrated a reduction in intranuclear $Ho_{342}$ content, up to a maximum of 35% of the level found in the parent as assessed by flow cytometry, despite similar levels of whole cell uptake determined using radiolabelled ligand. Ability to limit nuclear accessibility of $Ho_{342}$ correlated closely with cellular resistance to $Ho_{342}$ and to adriamycin. All drug resistant cell lines showed a significant increase in nuclear accessibility to $Ho_{342}$ after verapamil treatment, including a methotrexate resistant cell line. The methotrexate resistant variant, not demonstrating MDR, showed reduced nuclear binding of $Ho_{342}$ but increased cell kill associated with a propensity to develop a population of cells showing extra DNA replication in response to $Ho_{342}$ exposure. Differences between cell lines in the relationship between $Ho_{342}$-induced cell cycle perturbation and cell kill supported the conclusion that modulation of several pathways of response to cytotoxic agents had occurred in the development of drug resistance.

The clinical problem of acquired resistance to initially useful cytotoxic agents remains of major significance, and the investigation of cellular resistance mechanisms in tumour cell lines continues to generate hypotheses regarding this clinical situation. Prolonged exposure of mammalian cells *in vitro* to certain chemotherapeutic agents can result in the development of resistant sub-lines with cross-resistance to a number of functionally unrelated drugs. Such 'classical' multi-drug resistance (MDR) typically affects responsiveness to the anti-cancer drugs adriamycin (ADM), vincristine (VCR) and colchicine (COL). The MDR phenotype is thought to reflect increased active cellular efflux of these agents by a membrane located, energy-dependent transport mechanism involving P-glycoprotein (Gerlach *et al.*, 1986; Endicott & Ling, 1989). Presumably the mechanism of classical MDR effectively protects important intracellular targets by limiting their accessibility to these cytotoxic agents, an explanation satisfactory for the mitotic spindle inhibitors VCR and COL, but the situation is less clear for ADM, where significant cell membrane effects (Tritton & Yee, 1982) have been identified, in addition to DNA damaging activity (Zwelling *et al.*, 1981). Ideally an evaluation of intracellular target protection afforded by MDR should involve the use of an agent known to exert its cytotoxic effects predominantly through interaction with a single target (e.g. DNA), and whose delivery to this target can be monitored in individual cells.

We have investigated the relationship between cytotoxicity and the cellular capacity for intracellular target protection in cells showing MDR using a DNA specific ligand, Hoechst dye number 33342 (referred to as $Ho_{342}$; Figure 1). $Ho_{342}$ is a bisbenzimidazole dye with a specificity for non-intercalative binding at AT base pairs in DNA and minimal interaction with RNA (Latt & Stetten, 1976). The related dye Hoechst 33258 (see Figure 1) has been used extensively as a DNA stain (Zimmer & Wahnert, 1986). The more lipophilic derivative $Ho_{342}$ is used as a vital nuclear stain in flow cytometry (Latt, 1979) but can induce DNA damage, mutations and cell death (Durand & Olive, 1982). Human cells with putative deficiencies in DNA repair showed enhanced sensitivity to $Ho_{342}$ (Smith, 1984) and a mammalian cell mutant with enhanced capacity to remove $Ho_{342}$ from cellular DNA is highly resistant to the cytotoxic action of the dye (Smith *et al.*, 1988). Thus the interaction of $Ho_{342}$ with DNA appears to be an important factor in the cytotoxicity seen at higher concentrations of this agent (Zimmer & Wahnert, 1986).

**Figure 1** Chemical structures of selected bisbenzimidazole dyes and anthracyclines.

Unlike DNA-binding chemotherapeutic agents such as the anthracyclines (e.g. doxorubicin and daunomycin; Figure 1), the interaction of $Ho_{342}$ with its target (the nucleus) can be assessed by exploiting the considerable fluorescence enhancement that occurs when bisbenzimidazole dyes bind non-covalently to the minor groove of the DNA double helix (Latt & Stetten, 1976); using flow cytometry (FCM), the resulting $Ho_{342}$-DNA fluorescence monitors specifically nuclear dye uptake (Smith *et al.*, 1985; Morgan *et al.*, 1989). The FCM technique also allows the effects of resistance-modifying agents (verapamil, calmodulin inhibitors and $Ca^{2+}$ channel blockers; Tsuruo *et al.*, 1982) to be quantified and the kinetics of their actions defined (Krishan, 1987; Morgan *et al.*, 1989; Nooter *et al.*, 1989).

The present study examines the relationship between target protection and resistance in a series of murine cell lines

Correspondence: P.J. Smith.
Received 4 October 1989; and in revised form 23 July 1990.

initially selected for resistance to single anti-tumour agents (namely: ADM, COL, methotrexate (MTX), VCR, or a novel anthracycline, Roche product number 31-1215 (1215; Twentyman *et al.*, 1986*b*). The nuclear binding and cross-resistance patterns of the cell lines used, investigated by clonogenic assay, demonstrated participation of $Ho_{342}$ in the classical MDR pathway. The cytotoxicity study also incorporated a detailed analysis of $Ho_{342}$ cell cycle perturbation in an attempt to identify different cellular pathways for the expression of drug-induced cell killing in drug resistant cells.

### Materials and methods

### *Cell culture*

The derivation of cytotoxic drug resistant variants from the parent mouse mammary tumour cell line, EMT6/Ca/VJAC, has been described previously (Twentyman *et al.*, 1986*a*). Briefly cells from the parent cell line were continuously exposed to the cytotoxic agent to which resistance was being induced; over approximately 6 weeks the cytotoxic drug concentration, chosen such that continued cell growth occurred, could be progressively increased until ultimately the resulting cell line was able to grow in a drug concentration highly inhibitory to the parent cell line. Variant cell lines showing different degress of resistance to the inducing agent have been produced by a continuation of the same process; for this study variant cell lines were used which had closely similar cell cycle characteristics (Table I).

Cell lines were maintained in Eagles MEM with 20% new-born calf serum, supplemented with 2 mM glutamine, 100 IU $ml^{-1}$ penicillin and 100 mg $ml^{-1}$ streptomycin (all Gibco Biocult Ltd, Uxbridge, UK), at 37°C in 8% $CO_2$ in air. EMT6 lines grew as attached monolayers, and were detached for experiments using a short exposure to trypsin/EDTA in phosphate buffered saline (PBS).

### *Drug treatments*

The exact concentrations of filter sterilised stock solutions of $Ho_{342}$ (CP Laboratories, Bishop's Stortford, UK; Figure 1) were determined spectrophotometrically (molar extinction coefficient $4.1 \times 10^4$ $M^{-1}$ $cm^{-1}$ at 340 nm, pH 7.0). Adriamycin (Farmitalia Carlo Erba, St Albans, UK; Figure 1) was stored at −20°C at 500 mg $ml^{-1}$ in distilled water. Where indicated cells were exposed to VPL (Abbott Laboratories, Queenborough, UK) at 3.3 or 6.6 μM for 30 min at 37°C before addition of $Ho_{342}$. Cells were exposed to cytotoxic agents for 24 h under normal culture conditions (37°C, 92% air/8% $CO_2$, culture medium as above).

### *$Ho_{342}$ accumulation*

*Nuclear fluorescence assessment* Attached cells in logarithmic phase of growth ($1-3 \times 10^6$ per 9 cm dish), were exposed to fluorochrome under standard culture conditions at 37°C for 60 min before washing with buffer (10 mM Tris HCl, 100 mM NaCl, 10 mM EDTA, 1 mg $ml^{-1}$ bovine serum albumin, pH 8.0) at 4°C and rapid freezing on a dry ice/ethanol bath; immediately before FCM analysis cells were thawed at 37°C, resuspended in buffer at $2 \times 10^5$ cells $ml^{-1}$, and analysed using the MRC flow cytometer as described previously (Morgan *et al.*, 1989), importantly sub-cellular debris and cell clumps were excluded from analysis by electronic gating on the basis of light-scatter signals and pulse-shape analysis (Watson *et al.*, 1985). Fluorescence excitation was by a krypton laser tuned to 337 nm (200 mW light power) recording three parameters: 90° light scatter (<370 nm) acting as the master trigger, forward light scatter and fluorescence at 500 nm ± 5 nm wavelength, the median fluorescence value for whole cell populations was determined.

*Radiolabelled $Ho_{342}$ accumulation* Cells were treated in 96-well plates, under normal cell culture conditions, with $^3H$-$Ho_{342}$ (obtained through the tritium labelling service of NEN Research Products; supplied at a specific activity of 385 mCi $mmol^{-1}$), the nominal concentration of which ranged from 10 mM to 10 μM. After 60 min cells were washed twice with PBS at 4°C, allowed to dry in air, and lysed in 0.5 ml of 0.1 M NaOH for 60 min; aliquots were neutralised and counted in scintillation fluid (Aquasol). Assays were performed in triplicate; two plates set up in parallel were trypsin/EDTA detached for counting of total cell numbers and results expressed as radioactivity per $10^5$ cells. Radiochemical purity of $Ho_{342}$ was determined by thin layer chromatography of triethylamine-treated samples using 20% methanol in dichloromethane. The dry chromatogram was developed by applying 5 μl volumes of calf thymus DNA (1 mg $ml^{-1}$ PBS) along the trace and examining under long wave UV-illumination. Similar traces were observed for radiolabelled and unlabelled $Ho_{342}$ with greater than 40% of radioactivity associated with a constituent that showed fluorescence enhancement with DNA, the remaining activity not being associated specifically with any other short UV-radiation-absorbing constituents.

### *Cytotoxic drug sensitivity testing*

*Cell cycle perturbation* Attached cells in logarithmic phase of growth were treated with cytotoxic agents in 6-well plates. Cultures were washed with PBS at 4°C immediately before trypsin/EDTA detachment, and cells were resuspended in full culture medium at a density of $2 \times 10^5$ $ml^{-1}$ before staining, using a rapid one-step DNA-staining technique for RNase digested cells using ethidium bromide (50 μg ethidium bromide $ml^{-1}$, 0.125% Triton X-100, ribonuclease 0.5 μg $ml^{-1}$; 1:8 dilution with cell suspension; 10 min at room temperature: Taylor & Milthorpe, 1980). Details of the analysis of cell suspensions by FCM, and the computer algorithm used to determine cell cycle distribution have been published previously (Watson *et al.*, 1987). Where cytotoxic treatment resulted in very pronounced disturbance of the normal cell cycle distribution, and virtual complete loss of the $G_1$ population, this algorithm could not be used satisfactorily; in such cases the frequency distributions were assessed by manual setting of gates according to the $G_1$- and $G_2$-DNA positions of the control sample (concordance of these two methods was in all cases checked at the lower cytotoxic drug concentrations).

**Table I** Characteristics of murine cell lines

| Cell line | Selecting drug[a] | Drug conc. (μg $ml^{-1}$) | % cells in cell cycle phase[b] $G_1$ | S | $G_2/M$ | Mean cellular DNA content[c] (pg $cell^{-1}$) | P-glycoprotein[d] | Plating efficiency[e] |
|---|---|---|---|---|---|---|---|---|
| EMT6/P | – | – | 47.5 | 39.6 | 12.9 | 15.0 | + | 62±4% |
| AR1.0 | ADM | 1.0 | 34.5 | 47.2 | 18.2 | 15.3 | ++++ | 69±3% |
| CR2.0 | COL | 2.0 | 36.7 | 44.8 | 18.5 | 15.3 | +++++ | 38±8% |
| MR1.0 | MTX | 1.0 | 42.3 | 38.5 | 19.2 | 15.8 | + | 50±7% |
| VR1.0 | VCR | 1.0 | 43.6 | 35.4 | 21.0 | 16.4 | +++ | 69±4% |
| 1215R | Ro 31-1215 | 0.1 | 38.5 | 44.3 | 17.3 | 15.4 | ++ | 54±4% |

[a]Continuous exposure of stock cultures. [b]Typical values for exponential phase culture. [c]Mean value for whole population, exponential phase cells, with reference to human blood leucocytes, see text. [d]Data from Reeve *et al.* (1989), Twentyman *et al.* (1990) and Twentyman and Reeve (unpublished data). [e]Arithmetic means (±s.e.) for 9–11 determinations.

*Clonogenic assay* Cells grown in the absence of cytotoxic agents for 48 h were trypsin/EDTA detached, plated in fresh medium at a density of $2 \times 10^2$ cells (for lower cytotoxic drug concentrations) or $2 \times 10^3$ cells (for higher concentrations) per 6 cm dish, and allowed to attach for 6–12 h before cytotoxic drug exposure. Following drug treatments cultures were washed twice with PBS and maintained in drug-free, complete culture medium for 10 days without further medium change, and assayed by counting of fixed, stained, colonies.

### Results

### *Cytotoxicity*

The cross-resistance patterns of the various cell lines were studied for ADM and $Ho_{342}$. Clonogenic assay of cell survival following a 24 h $Ho_{342}$ treatment (Figure 2 and Table II), demonstrated a dose-dependent cell kill with all cell lines. This was also observed with EMT6/P after acute, 1 h exposures to $Ho_{342}$ (data not shown) but the $Ho_{342}$ concentrations required were considerably higher (e.g. for EMT6/P a $D_{80}$ of $>10\,\mu M$ for a 1 h, compared to $0.03\,\mu M$ for a 24 h exposure). This led to problems of $Ho_{342}$ precipitation in the phosphate based buffers at the concentrations required for MDR cell lines. Thus, a 24 h exposure period to either ADM or $Ho_{342}$ was selected for assessment of clonogenicity.

Clonogenic assay of cell survival for the lines AR1.0 and CR2.0 following ADM treatment (Figure 3 and Table II) demonstrated similar and considerable degrees of resistance to ADM, with resistance factors at the 10% survival level ($RF_{10}$; see Figure 3 for definition) of 78 and 88 respectively. The cell lines VR1.0 and 1215R showed an intermediate degree of resistance, with $RF_{10}$ values of 36 and 18 respectively; the ADM resistance of MR1.0 was minimal, with an $RF_{10}$ of 1.2. The cytotoxic drug resistant cell lines also showed a range of sensitivities to $Ho_{342}$, the ranking of resistance levels of the cell lines showing MDR being AR1.0 > CR2.0 ≫ VR1.0 > 1215R. This was essentially the same as that found for resistance to ADM, for which the ranking was AR1.0 and CR2.0 ≫ VR1.0 > 1215R, demonstrating participation of $Ho_{342}$ in the MDR phenotype. The MR1.0 line, which does not have the features of classical MDR (Table I), and showed no significant abnormality in ADM sensitivity, showed increased sensitivity to the cytotoxic actions of $Ho_{342}$ compared to EMT6/P ($RF_{10}$ 0.24).

### *$Ho_{342}$ uptake study*

Ligand uptake in the six cell lines, as assessed by radiolabelled $Ho_{342}$ accumulation in the whole cell (Figure 4), showed a direct correlation with fluorochrome concentration (determined spectrophotometrically) over the range 10 nM to 10 μM (the maximum concentration limited by the ethanolic stock solution) for all cell lines. No significant differences in whole cell uptake were observed between the parent cell line (EMT6/P) and the resistant variants studied. At the highest $Ho_{342}$ concentration studied (10 μM) cellular dye loads were calculated to be no greater than approximately 40 pmol per $10^5$ cells. In contrast, nuclear fluorescence, indicating nuclear binding of the ligand, although also dependent on concentration of the fluorochrome, demonstrated a non-linear relationship, with clear differences between the maximal levels of fluorescence for the variant cell lines (Figure 5, open triangles indicating results in the absence of VPL). EMT6/P showed a rapid increase in fluorescence up to 5 μM $Ho_{342}$, followed by a much smaller increase in fluorescence between 5 and 10 μM $Ho_{342}$, suggesting saturation of $Ho_{342}$–DNA binding sites at higher ligand concentrations. Least fluorescence was shown by the ADM resistant and the COL resistant lines, AR1.0 and CR2.0, in which 10 μM $Ho_{342}$ exposure produced 35% and 42% respectively of the fluorescence produced in the parent cell line, the shape of the fluorescence/drug concentration curves suggesting failure to saturate $Ho_{342}$–DNA binding sites even at the highest fluorochrome concentration used. In the remaining three drug resistant lines, MR1.0, VR1.0 and 1215R, there was a reduction of fluorescence compared to that of the parent line, to between 80% and 90%. These differences could not be accounted for on the basis of variations in cell cycle phase distribution or cellular DNA content (Table I).

**Figure 2** Sensitivity of mouse tumour cell lines of $Ho_{342}$: clonogenic assay after 24 h exposure to drug; mean ± 1 s.e.m. of three experiments each in quadruplicate; survival curves generated by computer algorithm. Symbols: ● EMT6/P; ○ AR1.0; ▲ CR2.0; △ MR1.0; ■ VR1.0; □ 1215R.

**Figure 3** Sensitivity of mouse tumour cell lines to ADM: clonogenic assay after 24 h exposure to drug; symbols as in Figure 2, ± 1 s.e.m. of quadruplicate plates; results of single typical experiment; survival curves generated by computer algorithm; dashed line indicates equivalent position of EMT6/P survival curve on the smaller scale.

**Table II** Relative drug sensitivities of murine cell lines

| | *Adriamycin* | | | *Ho-33342* | | |
|---|---|---|---|---|---|---|
| *Cell line* | $RF_{80}$ | $RF_{50}$ | $RF_{10}$ | $RF_{80}$ | $RF_{50}$ | $RF_{10}$ |
| AR1.0 | 40 | 54.0 | 78.3 | 19.7 | 37.1 | – |
| CR2.0 | 107 | 89.2 | 88.1 | 16.7 | 13.2 | 7.76 |
| MR1.0 | 1.6 | 1.24 | 1.17 | 0.042 | 0.12 | 0.24 |
| VR1.0 | 30 | 31.7 | 35.8 | 9.7 | 6.1 | 2.4 |
| 1215R | 5.0 | 9.5 | 18.2 | 1.03 | 1.21 | 1.3 |

Summary of clonogenic assay results shown in Figures 2 and 3. The resistance factor (RF) refers to: (dose yielding $x$% survival for variant/dose yielding $x$% survival for EMT6/P) measured at 80, 50 and 10% survival levels. Cell lines are referred to by the selecting agent used, e.g. EMT6/AR1.0 as AR1.0.

**Figure 4** Whole cell uptake of $Ho_{342}$ as assessed by $^3H$-$Ho_{342}$ exposure for 60 min. Symbols as in Figure 2.

**Figure 5** Nuclear binding of dye assessed by FCM after exposure of whole cells to $Ho_{342}$ at various concentrations for 60 min: median fluorescence of whole population ($10^4$ cells analysed) expressed as a percentage of that of EMT6/P cells after 60 min treatment with $Ho_{342}$ 10 µM. Symbols: Δ control; 30 min to VPL at either 5 µM (□), or 2.5 µM (■).

The acute effect of VPL exposure on the binding of $Ho_{342}$ to DNA was evaluated in the parent and resistant cell lines. EMT6/P cells exposed to VPL for 30 min before the addition of $Ho_{342}$ showed a small increase (10%) of $Ho_{342}$–DNA fluorescence, seen at 5 µM $Ho_{342}$ concentration only (Figure 5). Again, the lack of enhancement by VPL of the fluorescence produced by exposure of EMT6/P to 10 µM $Ho_{342}$ suggested saturation of DNA binding sites at this higher concentration of fluorochrome. All resistant cell lines showed VPL responsiveness at 3.3 µM concentration, with little additional effect of increasing VPL concentration to 6.6 µM. VPL increased the nuclear binding of DNA to $Ho_{342}$ (nuclear fluorescence) in the resistant cell lines CR2.0 and MR1.0 to that of the parent line, exposed to $Ho_{342}$ in the absence of VPL; indeed VPL pre-treatment was able to increase the nuclear fluorescence of VR1.0 and 1215R to levels above that of the parent line (for example, VR1.0; 10 µM $Ho_{342}$ gives 115% the fluorescence of EMT6/P). In contrast the $Ho_{342}$–DNA fluorescence of the ADM resistant line, EMT6/AR1.0, increased with VPL, but not to that level shown by the parent line even with the higher concentration of VPL. We conclude that there are considerable differences between drug/nuclear target interaction in drug resistant cells detectable by $Ho_{342}$–DNA fluorescence, but not apparent if whole cell uptake is considered.

The expected ranking of cellular resistance, predicted from the cellular capacity to limit $Ho_{342}$–DNA interaction is AR1.0 > CR2.0 ≫ VR1.0 > 1215R > MR1.0 > EMT6/*P*; a comparison of this ranking with the survival data presented above shows a close correlation between resistance and the limitation of the $Ho_{342}$–DNA binding. However a clear exception to this correlation is the MR1.0 cell line, in which reduced nuclear availability of the fluorochrome compared to the parent cell line was demonstrated by FCM, but which showed increased sensitivity to $Ho_{342}$ assessed by clonogenic assay.

### *Cell cycle perturbation produced by cytotoxic agents*

Cell cycle perturbation of an asynchronously growing population was examined at 24 h after cytotoxic drug exposure. Preliminary studies (data not shown) indicated the sensitivity of EMT6/P to ADM by an increase in the number of cells in $G_2$/M ($G_2$ arrest) at ADM concentrations of less than 10 µM, while these concentrations produced no significant increase in $G_2$/M proportion in the MDR cell lines, AR1.0, CR2.0, VR1.0 and 1215R. MR1.0 showed only a small increase in the $G_2$/M proportion at the highest (10 µM) cytotoxic concentration (data not shown). Exposure to $Ho_{342}$ (Figure 6) produced two effects on the cell cycle distribution. Firstly there was an accumulation of cells in $G_2$/M phase, which occurred at lower $Ho_{342}$ concentrations in the lines EMT6/P and MR1.0 compared to AR1.0, CR2.0 and VR1.0 lines, indicating the greater sensitivity of EMT6/P and MR1.0; this did not occur to any significant degree in the 1215R line. Secondly, a population of cells with high levels of DNA (i.e. HD cells) developed, which was seen with all cell lines but occurred at the lowest cytotoxic concentrations, and to the greatest extent, with MR1.0. HD cells developed in the 1215R line even though there was no significant increase in the proportion of $G_2$/M cells. $G_2$ arrest, or HD cell appearance, was associated with a $G_1$ emptying in all cell lines suggesting the absence of a $G_1$/S phase block. However under these conditions, with a 24 h drug exposure, a net depletion of S phase was only observed for MR1.0 cells, with all other cell lines showing either no change, or a net gain in S phase.

### *Relationship of cell cycle perturbation to cell viability*

If modification of nuclear binding of $Ho_{342}$ by the variant EMT6 cell lines is the only influence on cytotoxicity of this agent, then a similar dose modification effect should be seen for both clonogenicity and cell cycle perturbation in drug resistant cell lines. However, there may also be underlying differences in the manner in which cytotoxicity is effected in drug resistant cells, which could lead to a divergence between the cytotoxic drug dose modification required for one mode of evaluation of cytotoxicity, compared to that for the other. We have considered the association between cytotoxicity, as indicated by reduction of cell viability (clonogenicity), and cell cycle perturbation (specifically, the arrest of cells in $G_2$/M

**Figure 6** Effect of 24 h exposure to $Ho_{342}$ on cell cycle distribution in mouse tumour cell lines: expressed as percentage change in the proportion of cells in each phase in untreated control samples; means ± 1 s.e.m. of three experiments; □ $G_1$, ◆ S, and ▲ $G_2/M$; additionally the appearance of a population (HD; ◇) with increased DNA content is shown together with the total recruitment of cells into $G_2/M$ and HD (■) populations.

and the generation of HD cells). Figure 7 shows how these two parameters correlate for each cell line at given $Ho_{342}$ concentrations. (Preliminary analyses indicated that $G_2$ arrest alone was a relatively insensitive indicator of cell kill in EMT6 cell lines unless the concomitant arrest of cells in the HD phase was also considered.) A simple correlation between cell kill and cell cycle arrest might be expected to result in all values distributed along a straight line, with distance from the origin indicating either higher drug concentration or greater cellular sensitivity; EMT6/P cells demonstrated such a relationship (Figure 7, continuous line). The AR1.0 cell line showed very similar distribution of results to EMT6/P, with a close association of cell cycle changes with cell kill up to 0.6 fractional cell kill. At high $Ho_{342}$ concentrations cell cycle changes for EMT6/P were less than that expected for the degree of cell kill, suggesting that cell arrest was occurring in all phases of the cell cycle at these concentrations of dye, an effect observed with other cytotoxic agents (Barlogie *et al.*, 1976). The other cell lines examined showed divergence from the results of EMT6/P and AR1.0; CR2.0 and VR1.0 showed less cell cycle change for the same range of fractional cell kill, whereas 1215R showed an intermediate response. Figure 7 also clearly shows the relative hypersensitivity of MR1.0 to $Ho_{342}$, with high proportions of cells arrested in $G_2/M$ and the development of HD cells.

### Discussion

We have demonstrated varying degrees of suppression of nuclear binding of a DNA interactive fluorochrome, $Ho_{342}$, in the drug resistant variants of a murine tumour cell line. In

**Figure 7** Comparison of the total recruitment into $G_2$/M and HD populations shown by cell cycle analysis of dye-treated cells (detailed in Figure 6) with cytotoxicity (detailed in Figure 2) expressed as fractional cell kill (1, fraction of surviving cells) at various $Ho_{342}$ concentrations (shown in Figures 2 and 6). Symbols as in Figure 2.

those cell lines that showed both cross-resistance to ADM and changes associated with classical MDR (Table I), the ranking of DNA target protection (reduction in intranuclear $Ho_{342}$ content) was the same as both the ranking of cellular resistance to $Ho_{342}$ (AR1.0 > CR2.0 ≫ VR1.0 > 1215R), and the ranking of resistance to ADM (AR1.0 and CR2.0 ≫ VR1.0 > 1215R). These findings extend, to the murine cell lines examined, previous observations (Lalande *et al.*, 1981; Morgan *et al.*, 1989) that the cellular drug efflux mechanism of classical MDR operates on $Ho_{342}$. The degree of nuclear target protection from $Ho_{342}$ was shown to correlate closely with the degree of cellular resistance to a DNA interactive drug in the variant cell lines showing MDR. The effect of VPL in reversing the nuclear exclusion of $Ho_{342}$ was greatest on those cell lines showing greatest P-glycoprotein hyper-expression (AR1.0, CR2.0). However, in VR1.0 and 1215R, VPL increased $Ho_{342}$ uptake to levels above that of EMT6/P, suggesting that the differing capacity for drug efflux in MDR cell lines may mask underlying differences in DNA accessibility, which can be revealed by maximal blocking of the efflux mechanism.

The use of radiolabelled $Ho_{342}$ to monitor whole cell uptake did not indicate differences between the cell lines revealed by the fluorescence technique for intranuclear $Ho_{342}$ content. Reasons for this difference may include significant cell surface binding of radiolabelled $Ho_{342}$ or some masking effect of the background radioactivity not associated with DNA binding ligand (see Materials and methods). In separate studies (Coley *et al.*, 1989*a*,*b*; Twentyman *et al.*, 1990) it has been noted that the AR1.0, CR2.0, and VR1.0 cell lines show significant reductions in the whole cell accumulation of a radiolabelled anthracycline daunomycin and that these cell lines are also responsive to verapamil for the partial reversal of resistance to ADM. It is possible that the anthracyclines and the bisbenzimidazole dyes differ in their intracellular disposition and sequestration. In the case of the MDR cell lines, a second, intra-cellular verapamil-sensitive barrier limiting the access of fluorochrome to the nucleus may operate for $Ho_{342}$. Whether such a second level of control is associated with a P-glycoprotein-like efflux mechanism can only be speculated upon although subcellular fractionation studies would help to clarify this possibility. Given the dependence of $Ho_{342}$ upon DNA-interaction to effect cytotoxicity (see Introduction), we suggest that the flow cytometric method is a more appropriate method for detecting resistance to $Ho_{342}$. Furthermore, the findings are pertinent to those MDR drugs that may require access to discrete nuclear targets (e.g. topoisomerase poisons such epipodophyllotoxins and aminoacridines; Glisson & Ross, 1987).

An unexpected finding was the reduced nuclear accessibility of $Ho_{342}$ in MR1.0 cells, since P-glycoprotein hyper-expression (i.e. above the basal level found in EMT6/P) has not been demonstrated in this cell line (Table I), and resistance selection involved an agent, MTX, not typically involved in classical MDR. Moreover the whole cell uptake of tritiated daunomycin was not found to be reduced in this cell line (P.R. Twentyman, unpublished data). Although showing a smaller reduction in intranuclear content of $Ho_{342}$ than any other of the resistant lines test, there was a clear response of the MR1.0 line to the resistance modifier VPL, which increased the fluorescence to that of the parent, EMT6/P, line. There appears to be a range of expression of the *mdr1* gene in normal and tumour tissues (Fojo *et al.*, 1987; Goldstein *et al.*, 1989; Endicott & Ling, 1989), and it has been suggested that gene amplification or hyper-expression may reflect a non-specific 'stress response' of the cell. The induction of a VPL sensitive mechanism controlling nuclear accessibility to $Ho_{342}$ in the MR1.0 cell line could be a non-specific response of these cells to the stress of drug selection without functional significance in the mechanism of resistance to MTX, but this does not explain absence of detectable P-glycoprotein hyper-expression. It is also possible that VPL has activity on an alternative pathway in such drug resistant cells. We note that although the median fluorescence values quoted may have obscured the presence of a sub-population of VPL-sensitive cells showing highly modified $Ho_{342}$ uptake, this was not apparent upon analysis of the FCM data that showed a single population of cells, with a narrow range of fluorescence values, for each of the cell lines examined.

The ranking of five of the cell lines (P > 1215R > VR1.0 > CR2.0 > AR1.0) on the basis of nuclear fluorescence intensity was exactly that found for $Ho_{342}$ sensitivity. However, nuclear uptake alone does not explain the enhanced sensitivity of MR1.0 to $Ho_{342}$ compared to EMT6/P despite a reduced nuclear content of the ligand in the MR1.0 cell line. There remains the possibility that differences in cytotoxicity result from non-uniform distribution of the ligand within the genome, with binding to more critical portions of the genome in particular cell lines causing toxicity that is not indicated by their total nuclear binding of $Ho_{342}$.

In an attempt to determine the nature of the cellular responses to $Ho_{342}$ in the various cell lines we have assessed the relationship between cell cycle perturbation assay and clonogenicity. Conventionally COL is used as an arresting agent to determine cell cycle delay in the absence of cell division. However, this stathmokinetic approach would have been complicated by the known resistance of some of these lines to COL. Accordingly, here we have monitored the relative changes in each cell cycle phase compared to the untreated cells. Although differences in cell cycle duration and the rate of $G_2$ recruitment might interfere with this method of assessment, no such differences were in fact observed when camptothecin (a topoisomerase type I inhibitor to which all of these cell lines show identical sensitivity and similar rates of recruitment into $G_2$ delay) was used as the arresting agent (S.A. Morgan *et al.*, unpublished data). Surprisingly, only AR1.0 showed a control response suggesting that $Ho_{342}$ induces cell death by qualitatively different means in the other cell lines.

Cell cycle analysis demonstrated the development of a hyperdiploid population of cells in all cell lines as a result of exposure to $Ho_{342}$. Again the qualitative difference in cellular responses to $Ho_{342}$ is highlighted by the unusual propensity of the MR1.0 cell line (given the reduced $Ho_{342}$ uptake in these cells) to develop hyperdiploid cells at low dye concentrations. This propensity may reflect a facility for DNA replication without cytokinesis that has been observed in MTX resistant variants of other cell lines (Schimke, 1986).

The present study shows that cell lines which differ in $Ho_{342}$ sensitivity do not always show the expected changes in cell cycle perturbation (Figure 7). Thus some MDR cell lines (e.g. CR2.0 and VR1.0) may express modifications in pathways which affect ligand sensitivity additional to the protection of cellular DNA by the rapid cellular efflux of ligand molecules (e.g. ability to arrest cell cycle progression following the accumulation of genomic damage). The use of a DNA-specific ligand to analyse the relationship between cytotoxicity and nuclear location has revealed a spectrum of responses in drug resistant cells not predicted from their known degrees of expression of the classical MDR phenotype. We conclude that MDR can involve the modification of more than one cellular pathway controlling cytotoxic drug responsiveness, and that the flow cytometric approach described here may be useful in assessing such characteristics in human tumour biopsies.

The authors gratefully acknowledge the support and encouragement of Professor N.M. Bleehen.

### References

BARLOGIE, B., DREWINKO, B., JOHNSTON, D.A. & FREIREICH, E.J. (1976). The effect of adriamycin on cell cycle traverse of a human lymphoid cell line. *Cancer Res.*, **36**, 1975.

COLEY, H.M., TWENTYMAN, P.R. & WORKMAN, P. (1989*a*). Improved cellular accumulation is characteristic of anthracyclines which retain high activity in multidrug resistant cell lines, alone or in combination with verapamil or cyclosporin A. *Biochem. Pharmac.*, **38**, 4467.

COLEY, H.M., TWENTYMAN, P.R. & WORKMAN, P. (1989*b*). Identification of anthracyclines and related agents that retain preferential activity over Adriamycin in multidrug-resistant cell lines, and further resistance modification by verapamil and cyclosporin A. *Cancer Chemother. Pharmacol.*, **24**, 284.

DURAND, R.E. & OLIVE, P.L. (1982). Cytotoxicity, mutagenicity and DNA damage by Hoechst 33342. *J. Histochem. Cyctochem.*, **30**, 111.

ENDICOTT, J.A. & LING, V. (1989). The biochemistry of p-glycoprotein-mediated multidrug resistance. *A. Rev. Biochem.*, **58**, 137.

FOJO, A.T., UEDA, K., SLAMON, D.J., POPLACK, D.G., GOTTESMAN, M.M. & PASTAN, I. (1987). Expression of multi-drug resistance gene in human tumors and tissues. *Proc. Natl Acad. Sci. USA*, **84**, 265.

GERLACH, J.H., ENDICOTT, J.A., JURANKA, P.F. & 4 others (1986). Homology between P-glycoprotein and a bacterial haemolysin transport protein suggests a model for multidrug resistance. *Nature*, **324**, 485.

GLISSON, B.S. & ROSS, W.E. (1987). DNA topoisomerase II: a primer on the enzyme and its unique role as a multidrug target in cancer chemotherapy. *Pharmac. Ther.*, **32**, 89.

GOLDSTEIN, L.J., GALSKI, H., FOJO, A. & 11 others (1989). Expression of a multi-drug resistance gene in human cancers. *J. Natl Cancer Inst.*, **81**, 116.

KRISHAN, A. (1987). Effect of drug efflux blockers on vital staining of cellular DNA with Hoechst 33342. *Cytometry*, **8**, 642.

LALANDE, M.E., LING, V. & MILLER, R.G. (1981). Hoechst 33342 dye uptake as a probe of membrane permeability changes in mammalian cells. *Proc. Natl Acad. Sci. USA*, **78**, 363.

LATT, S.A. (1979). Fluorescent probes of DNA microstructure and synthesis. In *Flow Cytometry and Sorting*, Melamed, M.R., Mullaney, P.F. & Mendelsohn, M.L. (eds) p. 263. Wiley: New York.

LATT, S.A. & STETTEN, G. (1976). Spectral studies on 33258 Hoechst and related bisbenzimidazole dyes useful for fluorescent detection of deoxyribonucleic acid synthesis. *J. Histochem. Cytochem.*, **24**, 24.

MORGAN, S.A., WATSON, J.V., TWENTYMAN, P.R. & SMITH, P.J. (1989). Flow cytometric analysis of Hoechst 33342 uptake as an indicator of multi-drug resistance in human lung cancer. *Br. J. Cancer*, **60**, 282.

REEVE, J.G., WRIGHT, K.A., RABBITTS, P.H., KOCH, G.L.E. & TWENTYMAN, P.R. (1989). Collateral resistance to verapamil in multidrug-resistant mouse tumour cells. *J. Natl Cancer Inst.*, **81**, 1588.

SCHIMKE, R.T. (1986). Methotrexate resistance and gene amplification: mechanism and implications. *Cancer*, **57**, 1912.

SMITH, P.J. (1984). Relationship between a chromatin anomaly in ataxia telangiectasia cells and enhanced sensitivity to DNA damage. *Carcinogenesis*, **5**, 1345.

SMITH, P.J., LACEY, M., DEBENHAM, P.G. & WATSON, J.V. (1988). A mammalian cell mutant with enhanced capacity to dissociate a bis-benzimidazole dye-DNA complex. *Carcinogenesis*, **9**, 485.

SMITH, P.J., NAKEFF, A. & WATSON, J.V. (1985). Flow-cytometric detection of changes in the fluorescence emission spectrum of a vital DNA specific dye in human tumour cells. *Exp. Cell Res.*, **159**, 37.

TAYLOR, I.W. & MILTHORPE, B.K. (1980). An evaluation of DNA fluorochromes, staining techniques, and analysis for flow cytometry. *J. Histochem. Cytochem.*, **28**, 1224.

TRITTON, T.R. & YEE, G. (1982). The anticancer agent adriamycin can be actively cytotoxic without entering cells. *Science*, **217**, 248.

TSURUO, T., IIDA, H., TSUKAGOSHI, S. & SAKURAI, Y. (1982). Increased accumulation of vincristine and adriamycin effects in drug-resistant tumour cells following incubation with calcium antagonists and calmodulin inhibitors. *Cancer Res.*, **42**, 4730.

TWENTYMAN, P.R., FOX, N.E., WRIGHT, K.A. & 4 others (1986*a*). The *in vitro* effects and cross-resistance patterns of some novel anthracyclines. *Br. J. Cancer*, **53**, 585.

TWENTYMAN, P.R., FOX, N.E., WRIGHT, K.A. & BLEEHEN, N.M. (1986*b*) Derivation and preliminary characterisation of adriamycin resistant lines of human lung cancer cells. *Br. J. Cancer*, **53**, 529.

TWENTYMAN, P.R., REEVE, J.G., KOCH, G. & WRIGHT, K.A. (1990). Chemosensitization by verapamil and cyclosporin A in mouse tumour cells expressing different levels of P-glycoprotein and CP22 (sorcin). *Br. J. Cancer*, **62**, 89.

WATSON, J.V., CHAMBERS, S.H. & SMITH, P.J. (1987). A pragmatic approach to the analysis of DNA histograms with a definable G1 peak. *Cytometry*, **8**, 1.

WATSON, J.V., SIKORA, K.E. & EVANS, G.I. (1985). A simultaneous flow cytometric assay for c-*myc* oncoprotein and cellular DNA in nuclei from paraffin embedded material. *J. Immunol. Methods*, **83**, 179.

ZIMMER, C. & WAHNERT, U. (1986). Non intercalating DNA-binding ligands: specificity of the interaction and their use as tools in biophysical, biochemical and biological investigations of the genetic material. *Prog. Biophys. Molec. Biol.*, **47**, 31.

ZWELLING, L.A., MICHAELS, S., ERICKSON, L.C., UNGERLEIDER, R.S., NICHOLS, M. & KOHN, K.W. (1981). Protein-associated deoxyribonucleic acid strand breaks in L1210 cells treated with the deoxyribonucleic acid intercalating agents 4′-(9-acridinylamino) methanesulfon-*m*-anisidide and adriamycin. *Biochemistry*, **20**, 6553.